# Comparison of transcription of the *Haemophilus influenzae* iron/heme modulon genes *in vitro* and *in vivo* in the chinchilla middle ear

**DOI:** 10.1186/1471-2164-14-925

**Published:** 2013-12-27

**Authors:** Paul W Whitby, Timothy M VanWagoner, Thomas W Seale, Daniel J Morton, Terrence L Stull

**Affiliations:** 1 Department of Pediatrics, University of Oklahoma Health Sciences Center, Oklahoma City 73104, OK, USA; 2 Department of Biology, Oklahoma Christian University, Oklahoma City, OK 73136, USA; 3 Department of Microbiology and Immunology, University of Oklahoma Health Sciences Center, Oklahoma City 73104, OK, USA

**Keywords:** *Haemophilus influenzae*, Iron, Heme, Transcription

## Abstract

**Background:**

*Haemophilus influenzae* is a significant cause of childhood otitis media, and also has an absolute growth requirement for heme. Recent microarray studies using three *H. influenzae* isolates were used to propose a putative core of genes responsive to iron and heme levels. Included in the core modulon were thirty seven genes that are preferentially expressed under iron/heme limitation, most of which are directly involved with iron and or heme acquisition. In this report, the core iron/heme modulon was further refined following microarray analysis of two additional nontypeable *H. influenzae* isolates from patients with otitis media. The transcriptional status of the genes comprising the refined iron/heme core modulon was then assessed *in vivo*, in a chinchilla model of otitis media. These *in vivo* experiments were performed to address the hypothesis that iron and heme regulated genes are both highly expressed *in vivo* and important, during clinical infection.

**Results:**

Microarray analysis of two additional *H. influenzae* strains resulted in the definition of a core of iron/heme responsive genes. This core consisted of 35 genes maximally expressed under heme restriction and a further 20 genes maximally expressed in heme replete conditions. *In vivo* studies were performed with two nontypeable *H. influenzae* strains, 86-028NP and HI1722. The majority of operons identified as members of the core modulon by microarray were also actively upregulated in the chinchilla ear during otitis media. In 86-028NP, 70% of the operons were significantly upregulated while in HI1722 100% of the operons were upregulated in samples recovered from the chinchilla middle ear.

**Conclusion:**

This study elucidates a conserved core of *H. influenzae* genes the transcription of which is altered by the availability of iron and heme in the growth environment, and further assesses transcription of these genes *in vivo*. Elucidation of this modulon allows for identification of genes with unrecognized roles in iron/heme acquisition or homeostasis and/or potential roles in virulence. Defining these core genes is also of potential importance in identifying targets for therapeutic and vaccine designs since products of these genes are likely to be preferentially expressed during growth in iron/heme restricted sites of the human body.

## Background

The human body has evolved multiple mechanisms to provide nutritional immunity including those involved in sequestration of extracellular iron and heme. Bacterial pathogens have co-evolved multiple mechanisms to overcome these defenses and to utilize the sequestered micronutrients. *Haemophilus influenzae* is a human-specific pathogen that commonly resides as a commensal of the nasopharyngeal cavity. *H. influenzae* can ascend the eustachian tube and cause otitis media (OM) in children, can spread to cause disease in the lungs of patients with COPD and cystic fibrosis, and also can cause invasive disease [1-3]. These multiple environments inhabited by *H. influenzae* are all likely to be iron and heme (FeHm) restricted and are likely to differ in the iron and/or heme sources available to colonizing or invading bacteria. To overcome this FeHm restriction and utilize the multiplicity of possible FeHm sources, *H. influenzae* has evolved a complex array of mechanisms that specifically target iron- or heme-containing proteins as sources of these essential micronutrients [4-9].

*H. influenzae* has an absolute growth requirement for heme. The requirement is specifically for the immediate heme precursor, protoporphyrin IX (PPIX), since most strains possess the enzyme ferrochelatase [10]. In the human host, there is essentially no free PPIX, and the potential sources of heme are intracellular, in the form of hemoglobin or heme-containing enzymes such as the cytochromes, that are unavailable to invading microorganisms [11-13]. Hemoglobin released by lysis of erythrocytes is avidly bound by the serum protein haptoglobin, and the hemoglobin-haptoglobin complex is rapidly cleared from the circulation by hepatocytes [11,14]. Free heme, principally derived from the breakdown of methemoglobin, is bound by either of the serum proteins hemopexin or albumin and cleared from the circulatory system by hepatocytes [11,13]. Utilization of hemoglobin, the hemoglobin-haptoglobin complex, heme-hemopexin and heme-albumin complexes by *H. influenzae* requires a functional *tonB* gene indicating that uptake of heme from these host sources is mediated by TonB-dependent outer membrane proteins (TD-OMPs) [15-17]. An additional source of iron is serum ferri-transferrin, the utilization of which is mediated by the TD-OMP TbpA [18,19]. Previous studies have characterized both the potential human sources of iron and heme, including transferrin, hemoglobin, hemoglobin-haptoglobin, and the heme-hemopexin and heme-albumin complexes [4-6], as well as many of the OMPs mediating their utilization. These include the hemoglobin/hemoglobin-haptoglobin-binding TD-OMPs (Hgps) [20-22], theheme-utilization TD-OMP (Hup) [23], and the heme-hemopexin TD-OMP (Hxu) are regulated by the environmental availability of FeHm, and are preferentially transcribed under FeHm-restricted growth conditions [24,25].

Previously, we characterized the transcriptional changes that occur upon transition from a FeHm-starved to a FeHm-replete environment in three unrelated fully sequenced *H. influenzae* isolates, the extensively passaged laboratory strain Rd KW20, the type b *H. influenaze* (Hib) strain 10810 and the invasive non-typeable (NTHi) strain R2866 [24,25]. These data established that Rd KW20 had 162 genes responding whereas the two clinical isolates had significantly more such genes (respectively strains 10810 and R2866 had 351 and 363 genes). Transcription of 74 genes was altered by FeHm levels in all three strains. In addition, each isolate had several genes whose regulation was unique to that isolate. Rd KW20 had 15 genes that were upregulated by FeHm restriction that were present, but not regulated, in the other isolates. Strain 10810 had 78 and R2866 had 35 such genes. Each isolate also had a subset of genes that were both distinct to that isolate and responsive to FeHm levels. Rd KW20 had a single unique gene upregulated during FeHm restricted growth. In contrast, 10810 had 39 and R2866 had 12 similarly unique and regulated genes [25]. Overall, strains 10810 and R2866 more closely resembled one another in their transcriptional profiles than either resembled strains Rd KW20. Taken together, these data demonstrate that substantial heterogeneity exists among the individual FeHm-responsive modulons of these *H. influenzae* isolates. Nevertheless, these studies allowed us to define a core modulon of FeHm-responsive genes, numbering 74, that were responsive to alterations in FeHm levels in all three studied *H. influenzae* isolates.

The current study had two specific goals: 1) to further evaluate the FeHm-core regulon of *H. influenzae* by characterizing the *in vitro* transcriptomes of two additional NTHI strains, 86-028NP and R2866 which were isolated from the nasopharynx and the middle ear, respectively, of children with OM.; 2) to assess whether the regulation of gene transcription by FeHm levels *in vitro* accurately reflects the transcriptional status of the same genes *in vivo* in an animal model of acute OM.

## Results

### Defining the FeHm window of NTHi 86-028NP and R2846 for microarray analysis

Previously the growth conditions required to establish the FeHm-responsive genes of three *H. influenzae* strains were carefully defined [24,25]. In these previous studies the growth parameters included preincubation of the primary inoculum in growth medium containing heme at a concentration of 0.1 μg/ml prior to inoculation of the experimental culture. Initially the same growth protocol was used in the transcriptional analysis of isolates 86-028NP and R2846 reported herein. Under these conditions both isolates lost viability during the experimental growth period and the RNA yields from each were significantly decreased compared to the previous isolates studied. In previous experiments the *H. influenzae* isolates Rd KW20, R2866 and 10810 showed a continuous growth over the period of the experiment [24,25]. In contrast, NTHi 86-028NP showed a similar growth profile to approximately 90 minutes, with peak CFU values of 4 x10^7^ (+/- 9.2 x10^6^) but displayed a subsequent decrease thereafter to 2.8 X 10^7^ (+/- 3.5x 10^6^) CFU at 150 minutes. Isolate R2846 showed a similar profile to 86-028NP. RNA samples at 90 and 110 minutes from various experimental replicates showed varying yields of both total RNA and specific target mRNA, which resulted in a failure of the Q-PCR to analyze the potentially FeHm regulated genes. Thus, a systematic approach was taken to determine optimal conditions for analysis of *in vitro* gene transcription of these isolates. It seemed possible loss of viability of the isolates resulted from a deficiency of heme in the primary inoculum. As a result, a range of heme concentrations in the preincubation step were tested. When the inoculum culture was incubated at 2 μg/ml heme, both isolates retained viability across the period of the experiment and consistent transcriptional data was obtained. Using a set of test genes that included *hitA, tbp1, hxuC* and *ompP2*, we found that the kinetics of FeHm-mediated transcriptional regulation were similar to those observed for the three previously studied *H. influenzae* strains (data not shown).

### Microarray analysis of isolates NTHi 86-028NP and R2846

A microarray chip was designed that contained all the coding regions of the NTHi isolates 86-028NP and R2846. The same chip had been previously utilized to study the FeHm modulons of the Hib strain 10810 and the NTHi strain R2866 [25]. Triplicate cultures of each isolate were prepared and sampled as previously described [25].

Approximately 50–60 μg total RNA was purified from each experimental sample and submitted to NimbleGen Inc., where the samples underwent in-house quality control prior to microarray analysis. Of the 1820 ORFs on the array corresponding to NTHi 86-028NP genes, 64 (3.5%) were differentially transcribed in a statistically significant manner (Additional file 1: Fold transcriptional change of 86-028NP genes following supplementation of FeHM-restricted media with exogenous FeHm). Of these 64 genes, 39 (61%) were preferentially transcribed in FeHm-restricted conditions and 25 (39%) were maximally transcribed in FeHm-replete conditions. Of the 1664 ORFS represented on the array that correspond to NTHi R2846 genes, 94 (5.6%) were significantly differentially transcribed. Of these 94 genes, 70 (74%) were maximally transcribed in FeHm-restricted conditions and 24 (26%) in FeHm-replete conditions (Additional file 2: Fold transcriptional change of R2846 genes following supplementation of FeHM-restricted media with exogenous FeHm). In both sets of microarray data, transcripts of the known FeHm-repressible genes *tbp1*, *hitA* and *hxuC* were elevated under FeHm restriction while transcripts of the constitutive gene *ompP2* showed no significant changes in either isolate. These findings were in accordance with the preliminary quantitative PCR (Q-PCR) experiments. [For simplicity, genes preferentially transcribed in FeHm-restricted media or FeHm-replete media will now be referred to as FeHm negative (FeHm-ve) or FeHm positive (FeHm + ve) respectively]. In comparison with the previous microarray studies, the number of genes responding to altered FeHm status is lower in both 86-028NP and R2846 than was observed for the previously reported isolates. This discrepancy may reflect the resulting effect of the additional heme required in the initial growth of the inocula cultures for these isolates.

### Validation of the microarray data

Several genes were selected for analysis by Q-PCR to validate the microarray data. A separate experiment was performed for each isolate to purify RNA from FeHm-deplete and -replete cells using the same conditions as those described for the microarray analysis. Genes analyzed for each isolate included ten examples of genes preferentially expressed under FeHm limitation or supplementation. We observed concordance of 100% between the Q-PCR and the microarray transcription data (Table 1).

**Table 1:** Q-PCR validation of Microarray Results

**Fold Change**^**a**^				
	**R2846**		**86-028NP**	
**Locus**^**b**^	**Microarray**	**Q-PCR**	**Microarray**	**Q-PCR**
HI0075	-2.3	-6.4	-1.7	-2.8
HI0095	-2.2	-14.4	-3.5	-4.4
HI0097	-2.4	-68.0	-2.4	-4.0
HI0153	-2.0	-2.5	-1.5	-2.1
HI0185	12.2	2.3	2.9	6.5
HI0253	-1.6	-11.3	-1.8	-3.2
HI0994	-8.4	-7.6	-3.2	-5.2
HI1094	1.6	3.0	1.7	1.9
HI1369	-8.0	-10.0	-1.7	-8.3
HI1384	1.6	2.3	2.0	26.3

^a^Fold change in transcript levels between FeHm replete and deplete conditions.

^b^Gene locus in Rd KW20.

### Comparison of the microarray data of *H. influenzae* isolates 86-028NP, R2846, R2866, Rd KW20 and 10810

In an initial analysis of the FeHm-responsive genes of *H. influenzae,* the well-characterized laboratory strain Rd KW20 had 80 FeHm-ve genes and 82 FeHm + ve genes [24]. Addition of data from the Hib isolate 10810 and the NTHi isolate R2866, subsequently established a common core of 37 FeHm-ve and 37 FeHm + ve genes responding to FeHm availability in these 3 strains [25]. Combining that data with the newly acquired data from R2846 and 86-028NP now allows the further refinement of the definition of the core FeHm modulon for the species *H. influenzae*. We now define a gene as a member of this modulon if it is significantly regulated in at least 4 of the 5 isolates evaluated. In a few operons, one or two genes distal to the promoter may have fallen below the 1.5-fold inclusion limit but were included as part of the core if they were regulated in at least 3 isolates. The core FeHm modulon of the 5 isolates contains 35 genes that are preferentially transcribed under FeHm-deplete conditions and 20 genes that are preferentially transcribed under FeHm-replete conditions (Tables 2 and 3). Of the FeHm-ve genes the majority (20 genes; 57%) have a known or putative role in iron and/or heme uptake. Genes encoding the characterized heme-hemopexin uptake system (*hxuABC*), the transferrin uptake system (*tbp12*), the energy transducing TonBExbBExbD system and the inner membrane iron transporter HitABC were significantly upregulated in all five isolates. Several genes whose products are predicted to be directly involved in uptake of heme across the OM were also regulated in all strains. These include a probable heme uptake receptor HemR (HI0113 in strain Rd KW20), the TD-OMP designated HI1369 in Rd KW20, and OmpU (HI0997m in Rd KW20). Several other FeHm-related genes were also found to be regulated in four isolates. These include genes encoding the hemoglobin-haptoglobin receptors HgpB and HgpC and the putative inner membrane divalent cation transporter system YfeABCD.

**Table 2:** Loci preferentially expressed in 4 or more strains in FeHm-restricted conditions

		**Fold change**^**c**^				
**Locus**^**a**^	**Description**^**b**^	**R2866**	**10810**	**Rd**	**R2846**	**86-028NP**
HI0035	Conserved hypothetical protein/predicted transporter	-2.46	-1.74	-1.55	-2.16	*-1.19*
HI0075	Anaerobic ribonucleoside-triphosphate reductase, alpha subunit NrdD	-2.95	-2.54	-4.03	-2.34	-1.66
HI0095	Putative methyltransferase	-12.56	-3.93	-10.21	-2.15	-3.47
HI0097	Iron (III) ABC transporter, periplasmic binding protein HitA	-14.27	-4.52	-8.15	-2.39	-2.38
HI0098	Iron (III) ABC transporter, permease protein HitB	-1.80	-2.70	-3.55	-2.33	-2.24
HI0099	Iron (III) ABC transporter, ATP-binding protein HitC	-2.01	-2.16	-3.56	-2.19	-2.03
HI0113	Probable TonB-dependent heme receptor HemR	-4.72	-2.28	-2.36	-3.39	-1.64
HI0153	Putative anaerobic C4-dicarboxylate transporter	-2.70	-2.24	not on^d^	-2.00	-1.54
HI0251	Protein TonB	-1.99	-1.69	-2.75	*-1.42*	*-1.40*
HI0252	Biopolymer transport ExbD	-2.45	-1.87	-3.02	-1.54	-1.76
HI0253	Biopolymer transport protein ExbB	-3.07	-2.26	-2.69	-1.64	-1.84
HI0262	Heme-hemopexin utilization protein HxuC	-24.69	-4.03	-10.30	-3.95	-2.64
HI0263	Heme-hemopexin utilization protein HxuB	-29.31	-3.29	-9.20	-2.92	-3.93
HI0264	Heme-hemopexin utilization protein HxuA	-18.55	-2.86	-7.44	-2.55	-2.11
HI0359	Fe/Mn/Zn ABC transporter, permease protein YfeD	-2.92	ns	-4.35	*-1.31*	-1.66
HI0360	Fe/Mn/Zn ABC transporter, permease protein YfeC	-3.54	ns	-6.90	*-1.34*	-1.81
HI0361	Fe/Mn/Zn ABC transporter, ATP-binding protein YfeB	-7.89	ns	-8.91	-1.74	-2.12
HI0362	Fe/Mn/Zn ABC transporter, periplasmic binding protein YfeA	-6.01	*-1.17*	-11.74	-1.57	-1.78
HI0534	Aspartate-ammonia lyase AspA	-1.56	-3.38	-3.12	-2.62	-1.67
HI0584	Putative peptidase/hydrolase	-1.84	-1.59	-1.69	-1.75	ns
HI0661	Hemoglobin-haptoglobin binding protein HgpB	-6.52	-4.14	*-1.20*	-2.51	-3.09
HI0691	Glycerol kinase GlpK	-5.71	-3.40	-1.61	-1.58	ns
HI0712	Hemoglobin-haptoglobin binding protein HgpC	-9.82	-2.94	-6.64	-2.02	ns
HI0809	Phosphoenolpyruvate carboxykinase PckA	-6.41	-3.16	-2.87	-1.92	*-1.35*
HI0994	Transferrin-binding protein Tbp1	-9.56	-8.16	-12.40	-8.43	-3.15
HI0995	Transferrin-binding protein Tbp2	-19.45	-16.53	-13.94	-8.57	-6.92
HI0997m	Putative outer membrane protein OmpU	-15.68	-14.61	not on^d^	-9.24	-7.44
HI1210	Malate dehydrogenase Mdh	-5.29	-2.68	-2.47	-1.97	*-1.49*
HI1356	4-alpha-glucanotransferase (amylomaltase) MalQ	-2.32	-2.25	-1.82	-2.29	-1.76
HI1357	1,4-alpha-gulcan branching enzyme GlgB	-2.75	-2.67	-1.59	-2.44	-1.65
HI1358	Glycogen debranching enzyme GlgX	-2.38	-2.80	-1.51	-2.35	*-1.48*
HI1359	Glucose-1-phosphate adenyltransferase GlgC	-3.28	-3.10	-1.73	-2.32	*-1.44*
HI1360	Glycogen synthase GlgA	-2.03	-2.56	-1.52	-2.03	ns
HI1369	Probable TonB-dependent transporter	-10.65	-1.56	-7.02	-8.04	-1.70
HI1427	Putative ABC transport, periplasmic binding protein	-6.60	-2.32	-6.12	-2.65	-1.52

^a^Gene locus in Rd KW20.

^b^Name and description of the gene based upon annotation of Rd KW20 or R2846 and R2866.

^c^Fold change as determined from the microarray data. Numbers in italics are below the 1.5 fold threshold but have a p-value below the statistically significant threshold. Numbers in bold are above the 1.5 fold threshold but have a p-value above the statistically significant threshold. **ns** = non significant and below 1.5-fold threshold.

^d^locus not included on the Rd KW20 array but expression determined to be above 2 fold change in expression by Q-PCR.

**Table 3:** Loci preferentially expressed in 4 or more strains in FeHm-replete conditions

		**Fold change**^**c**^				
**Locus**^**a**^	**Description**^**b**^	**R2866**	**10810**	**Rd**	**R2846**	**86-028NP**
HI0006m	Formate dehydrogenase-N, alpha subunit FdnG	+7.58	+8.36	not on^d^	*+1.44*	+1.71
HI0007	Formate dehydrogenase-N, Fe-S beta subunit FdnH	+7.49	+6.00	+3.40	*+1.21*	+1.73
HI0008	Formate dehydrogenase-N, cytochrome B556 gamma subunit, FdnI	+7.47	+5.23	+3.20	ns	+1.76
HI0009	Formate dehydrogenase-N, accessory protein FdnE	+3.35	+3.43	+1.88	ns	+1.75
HI0185	Formaldehyde dehydrogenase, glutathione-dependent AdhC	+5.86	+4.01	+8.04	+12.18	+2.85
HI0343	Twin-arginine signal-peptide-binding chaperone NapD	+3.19	ns	+1.66	+1.78	+1.60
HI0344	Periplasmic nitrate reductase subunit NapA	+2.67	ns	not on^d^	+1.79	+1.62
HI0345	Periplasmic nitrate reductase, ferredoxin-type protein NapG	+4.47	ns	+1.88	+2.13	+1.91
HI0346	Periplasmic nitrate reductase, ferredoxin-type protein NapH	+3.37	*+1.25*	+1.93	+1.86	+1.71
HI0347	Periplasmic nitrate reductase, electron transfer subunit NapB	+4.77	ns	+1.90	+1.68	+2.17
HI0348	Periplasmic nitrate reductase, cytochrome C-type subunit NapC	+3.20	ns	+1.71	+1.69	+2.34
HI0980	DNA architectural protein Fis	+4.67	+2.56	+2.06	+1.66	*+1.15*
HI1066	Nitrite reductase complex, transmembrane protein NrfD	+6.34	+2.24	+1.88	+3.84	ns
HI1067	Nitrite reductase complex, Fe-S subunit NrfC	+6.99	+1.70	+1.52	+2.98	+3.53
HI1068	Nitrite reductase complex, periplasmic cytochrome subunit NrfB	+9.49	ns	+2.00	+4.75	+3.81
HI1069	Nitrite reductase complex, periplasmic cytochrome C552 subunit NrfA	+8.33	+2.53	+2.13	+5.11	+3.65
HI1078	Probable amino acid ABC transporter, ATP-binding protein	+1.88	+1.89	+1.52	+2.21	*+1.16*
HI1094	Cytochrome c-type biogenesis protein CcmF	+2.53	+1.60	+1.81	+1.63	+1.65
HI1384	Ferritin protein A1	+3.21	+2.01	+2.60	+1.62	+2.06
HI1385	Ferritin protein A2	+4.13	+2.24	+2.94	+1.58	+2.23

^a^Gene locus in Rd KW20.

^b^Name and description of the gene based upon annotation of Rd KW20, R2846 and R2866.

^c^ Fold change as determined from the microarray data. Numbers in italics are below the 1.5 fold threshold but have a p-value below the statistically significant threshold. Numbers in bold are above the 1.5 fold threshold but have a p-value above the statistically significant threshold. **ns** = non significant and below 1.5-fold threshold.

^d^locus not included on the Rd KW20 array but expression determined to be above 2 fold change in expression by Q-PCR.

Among the 20 core genes preferentially expressed under FeHm-replete conditions, only six were regulated in all 5 isolates. These include genes encoding the ferritin subunits (HI1384-HI1385) as well as a glutathione-dependent formaldehyde dehydrogense (HI0185), two components of the nitrate reduction complex (HI1067 and HI1069), and a cytochrome C biogenesis gene (HI1094).

### Comparison of the 5 genome core, with the previously described 3 genome core

A previous iteration of the FeHm-responsive core modulon of *H. influenzae* was based on microarray analyses of 3 isolates [25]. For the FeHm-ve gene set inclusion of the two additional strains reported herein resulted in the exclusion of 17 genes included in the previous 3-genome based core modulon and the addition of 14 genes that were previously excluded. None of the 17 genes that were removed from the FeHm-ve core are believed to be directly involved in FeHm acquisition. However, additions to the consensus FeHm-ve core include many genes with an established or putative role in FeHm homeostasis. One locus added to the FeHm-ve core as a result of the current study is the the *yfeABCD* locus (HI0361-HI0364 in Rd KW20). This locus was previously excluded since it is constitutively expressed in the Hib strain 10810, possibly as a result of a nucleotide substitution within a putative Fur box upstream of the gene [26]. The inclusion of the *yfeABCD* locus in the modified core reported herein attests to the value of multi-genome analysis for the definition of regulons within a species. Other genes added to the core included some within operons in which the first gene was previously reported as part of the 3-genome core.

An even greater degree of change was seen for the set of genes preferentially expressed under FeHm-replete conditions. Twenty five genes that were part of the 3-genome core were excluded from the 5-genome core while eight genes were added. Of the 8 added genes, six are members of the locus encoding components of the nitrate reductase complex, *napDAGHBC* (HI0343-HI0348 in Rd KW20). Another added gene was the promoter proximal gene of the *fdnGHIE* (HI0006m-HI0009 in Rd KW20) operon which encodes a nitrate-inducible formate dehydrogenase. This gene had been excluded from the previous Rd KW20 array although it was subsequently shown by Q-PCR to be regulated in that isolate. The last added gene was within the *nfrABCD* (HI1066-HI1069 in Rd KW20) operon, the other genes of which were already included in the core. These three operons include 14 of 20 genes (70%) included in the final FeHm + ve core.

There were 47 genes in 32 putative operons that did not exhibit significant changes in transcription in either R2846 or 86-028NP but were regulated by FeHm in the previous studies with Rd KW20, 10810 and R2866 [25]. Given the additional heme required to maintain viability in strains R2846 and 86-028NP, these genes may represent loci whose expression is only altered under extreme FeHm stress. Examination of these genes revealed no clear pattern of expression beyond a suggestion that the lower FeHm levels have more profoundly stressed the cells. Genes that are preferentially expressed in FeHm deplete conditions in the three original isolates include those that encode a protein that replaces an oxidatively-damaged pyruvate lyase subunit, several transporters and associated utilization proteins (predicted tricarboxylate, gluconate, tryptophan and uracil transporters), a periplasmic NAD nucleotidase involved in NAD and NAD(P) scavenging, a stationary phase translation inhibitor and a tRNA recycling protein, the Dps protein that protects DNA from oxidative damage, and several other enzymes. Among genes preferentially expressed following the return to FeHm-replete conditions, 12 of the 27 are genes whose products are involved in tRNA or ribosome maturation. This suggests that protein synthesis may have been suppressed during FeHm stress. The remaining genes have only putative assigned functions.

### *In vitro* examination of the FeHm core modulon in the clinical NTHi isolate HI1722

We were unable to utilize strain R2846 for *in vivo* studies since in preliminary experiments in the chinchilla infected animals rapidly developed symptoms of inner ear infection which represent criteria for termination of the protocol. As a result an alternate strain was selected for *in vivo* determination of transcription. The NTHi isolate HI1722 was chosen for these studies because it was isolated from a patient with OM and has been previously utilized in the chinchilla model of OM [27]. To facilitate the *in vivo* examination, this isolate was partially genome sequenced to determine the presence or absence of core FeHm-responsive genes and the sequence of individual genes to ensure homology of the Q-PCR primers. Similarly to the NTHi strains 86-028NP and R2846, strain HI1722 required preincubation with 2 μg/ml heme to prevent cell death over the time course of the regulation studies (data not shown). Initially the transcriptional response of the genes *tbp1*, *hxuC,* and *ompP2* were examined. Similar kinetics of expression were observed for each gene as those previously described for the five other isolates previously examined (data not shown).

The transcriptional profiles of the genes comprising the 5-genome FeHm-responsive core were examined in HI1722 by Q-PCR. For genes in operons, a single gene was chosen as representative of the operon. Of the 28 operons in the FeHm core 18 were regulated in this previously unstudied isolate including every operon that was FeHm responsive in all 5 previously studied isolates (Table 4). In addition, 7 genes unresponsive to FeHm levels in any of the previously studied strains were selected as control genes and the transcript levels determined for HI1722. Transcript levels of all 7 control genes did not change in response to FeHm levels in HI1722 (data not shown).

**Table 4:** Transcription of core FeHm-responsive genes in the NTHi isolates 86-028NP and HI1722

		**Fold Change**^**b**^			
		***86-028NP***		**HI1722**	
** Locus**^**a**^	** Gene**	***In vitro***	***In vivo***^***c***^	***in vitro***	***in vivo***^***d***^
HI0035		2.25	15.53	***1.07***	29.37
HI0075	*nrdD*	2.4	3.81	5.28	21.31
HI0095		9.69	7.11	29.61	22.01
HI0097	*hitA*	19.92	2.38	66.15	34.66
HI0113	*hemR*	1.68	2.47	3.02	5.82
HI0153	*dcuB*	1.96	7.17	***1.39***	5.57
HI0253	*exbB*	3.11	***-2.44***	9.02	37.74
HI0263	*hxuB*	5.97	5.95	11.91	14.19
HI0362	*yfeA*	3.14	2.17	19.93	81.46
HI0534	*aspA*	5.19	6.74	***-1.06***	36.7
HI0584		1.75	2.52	***1.03***	10.05
HI0661	*hgpB*	11.36	3.01	25.6	129.37
HI0691	*glpK*	***1.39***	***1.22***	***-1.11***	4.26
HI0809	*pckA*	2.48	***1.3***	1.51	29.87
HI0994	*tbp1*	8.73	3.65	15.66	28.3
HI0997m	*ompU1*	12.68	42.36	7.77	30.71
HI1210	*mdh*	2.11	2.58	***1.21***	3.5
HI1356	*malQ*	2.36	10.27	***-1.92***	10.86
HI1369		8.94	30.26	5.15	30.63
HI1427		3.63	8.8	3.33	7.42
HI0007	*fdnH*	-2.3	***1.15***	-3.84	***1.81***
HI0185	*adhC*	-4.48	***-1.42***	-5.35	***-1.42***
HI0343	*napD*	***-1.38***	***1.56***	***1.00***	***12.4***
HI0980	*fis*	***-1.05***	***1.26***	***-1.18***	***2.36***
HI1069	*nrfA*	-5.08	***2.28***	-5.06	***9.69***
HI1078	*tcyC*	-1.92	***2.58***	***-1.01***	***3.79***
HI1094	*ccmF*	-1.51	***9.74***	-2.01	***4.68***
HI1384	*ftnA*	-4.27	-7.88	-6.89	-5.41

^a^Gene locus in Rd KW20.

^b^Fold difference in gene expression levels in theFeHm-deplete *in vitro* sample when normalized to replete condition. Numbers in bold are data points that differ in fold change from that predicted by the core FeHm regulon.

^c^Geometric mean of 18 samples, normalized to heme-replete *in vitro* sample.

^d^Geometric mean of 6 samples, normalized to heme-replete *in vitro* sample.

### Transcriptional status of the core modulon of 86-028-NP and HI1722 during experimental OM in the chinchilla

A major goal of this study was to determine the *in vivo* transcriptional status of the core FeHm-modulon genes in the chinchilla middle ear a clinically relevant animal model of disease. To allow a direct comparison of transcript levels between the *in vivo* and *in vitro* derived samples, each was normalized to the time zero FeHm supplemented *in vitro* grown culture of the respective isolate. In this way the expression level determined by Q-PCR of the ear samples can be compared to the *in vitro* FeHm deplete/supplemented values since they are all normalized to the same internal “housekeeping” gene *gyrA*. In essence, the *in vitro* data provide two indices of transcriptional status, an upper level, corresponding to upregulation and a lower level corresponding to fully FeHm repressed basal transcription. For each gene in the FeHm core, the fold change in transcripts in response to FeHm addition *in vitro* had been determined (Tables 2, 3 and 4). Since a fully repressed sample is used as a normalizer, all the other values are “fold change with respect to full repression” thus positive numbers above 1.5 indicate increased transcription, values between +/- 1.5 are not considered indicative of a change and values below -1.5 are further repressed. To test the hypothesis that genes in the FeHm core are transcribed *in vivo*, cohorts of chinchillas were infected and ear effusion samples were collected at various times for determination of the transcriptional status of genes in the FeHm core modulon. Following Q-PCR of each gene of interest in each sample, a set of profiles of transcription for each gene was determined and compared to the *in vitro* data. Table 4 displays the Q-PCR results (geometric mean) for each gene in each isolate together with the data of the *in vitro* grown samples (normalized to heme-replete conditions). Individual data points for each gene, for each day are shown in Additional file 3: Q-PCR values for FeHm core genes in 86-028NP infected chinchilla ears and in Additional file 4: Q-PCR values for FeHm core genes in HI1722 infected chinchilla ears. For most genes, the *in vivo* level of transcripts was similar to, or in excess of, the *in vitro* data for FeHm-restricted conditions. Values are bolded where the transcripts of a gene showed either a fold change below our cut off (indicating non regulation) of a fold change opposite to that predicted by the *in vitro* FeHm regulon. In addition, the transcriptional status of the selected genes appears to be relatively stable across the duration of the experiment (Additional files 3 and 4), indicating that over the course of the experiment the middle ear fluids remained FeHm restricted. Several interesting observations were noted when the *in vivo* and *in vitro* data for the two isolates were compared. For genes observed to be preferentially expressed in FeHm-deplete conditions in the microarray studies, nearly all were expressed at a high level *in vivo*. However, many of the operons that were preferentially expressed in FeHm-replete conditions were also expressed at a higher level in the *in vivo* samples for both isolates. The only exceptions were the *adhC* and *ftnA* genes. A likely explanation for this is that the microenvironment may contain additional physiological signals such as nutrient depletion, redox stress and other such stimuli which may lead to expression of these genes in this environ. A second interesting observation is the low expression levels of *exbB* in the 86-028NP *in vivo* data (Table 4). This finding was confirmed by determination of the transcript levels of *tonB* in each of the 86-028NP ear samples. The results were consistent with the lower expression of *exbB* in the chinchilla ear (data not shown). For the HI1722 isolate, each of the core FeHm-ve modulon genes were significantly upregulated in the *in vivo* samples. Taken in its entirety, the data discussed in this section confirm that the chinchilla ear fluids remain FeHm-limited during the period of the experimental infection.

## Discussion

The two main goals of this study were to refine the core of FeHm-modulated genes for the species *H. influenzae* and assess the correlation of transcriptional profiles observed during experimental OM. In performing the *in vitro* studies it was clear that not all *H. influenzae* isolates are equally resistant to prolonged FeHm starvation. In previous studies using the three strains Rd KW20, 10810 and R2866, incubation of the seed culture with 0.1 μg/ml heme was sufficient to allow subsequent survival in FeHm-deplete media for at least a further two hours and reproducible gene transcript levels over time. However, for the three strains used in the present study (86-028NP, R2846 and HI1722) similar conditions led to rapid loss of viability of the isolates and poor reproducibility of transcript analyses. Titration of the heme in the preincubation period demonstrated that a minimum of 2 μg/ml heme was required to retain viability of these strains. The physiological reason for this phenomenon is unknown. Interestingly, the three isolates reported herein share a similar genetic trait distinct from the isolates in the first study. In each of the isolates there is the insertion of a FeHm-modulated hemoglobin-haptoglobin binding protein gene (*hgpA*) at a locus containing the polyamine genes, *potE* and *speF*, which replaces the latter two genes. In Rd KW20, 10810 and R2866 the *potE* and *speF* genes are also FeHm regulated. Thus, the lack of *potE* and *speF* may be responsible for the reported inability to survive protracted FeHm starvation. It is possible that the role of the polyamines in *potE/speF* containing strains is to provide a protective effect during fermentative growth as polyamines have been shown to protect against acid stress [28]. It has been previously shown that *H. influenzae* is able to grow fermentatively with the production of acids as a byproduct [29,30]. In the isolates lacking these genes, the ability to persist under fermentative growth would be limited. Thus, a higher initial concentration of heme may be required to provide a sufficient intra-cellular concentration to ensure adequate functional respiratory enzymes and allow respiration over the period of the experiment. Further studies are planned to investigate the role of the polyamine locus in long term resistance to FeHm starvation. Although *hgpA* and *potE/speF* share the same genetic location and both are maximally expressed under FeHm-depleted conditions, they have distinct upstream regions and are not regulated by the same promoter element (data not shown).

The individual *in vitro* FeHm modulons indicate that 86-028NP and R2846 are more similar to each other than they are to any of the three isolates characterized in our previous studies [24,25]. This may simply result from the need for additional heme required in the current studies. In the newly described modulons there are fewer regulated genes. This is most pronounced in the subset of genes preferentially expressed under FeHm-replete conditions. The majority of the differentially regulated genes appear to be involved in basic metabolism and include various transferases and ribosome-associated proteins. While these genes are regulated in Rd KW20, R2866 and 10810, the lack of regulation in R2846 and 86-028NP indicates a difference in the basic metabolism between these two sets of isolates. Specifically it suggests that the higher level of heme leads to less of an impact on central metabolism during FeHm depletion. Thus, the current study may more accurately reflect the genes directly regulated by FeHm availability as opposed to those for which the transcriptional status is secondary to the consequences of FeHm starvation.

Additionally the current study tested the hypothesis that genes preferentially expressed under conditions of FeHm starvation would be similarly upregulated in the middle ear during experimental infection. Two isolates that have been previously used in the chinchilla model were selected for these studies. In NTHi strain HI1722, each core gene that was upregulated in FeHm-deplete conditions *in vitro* was also apparently upregulated during experimental otitis media (Table 4). Combined with the observation that nearly all of the FeHm-ve genes in 86-028NP were also highly transcribed *in vivo*, these findings validate the hypothesis that *H. influenzae* genes preferentially transcribed under FeHm-limited growth are also transcribed in the middle ear during OM.

## Conclusions

The targeted examination of gene regulation in the chinchilla ear reported herein does not address all of the FeHm-regulated genes that may contribute to virulence in that environment for any specific isolate. Rather the study focused on the core FeHm-responsive genes; the microarray studies clearly show that each of the isolates contained additional genes that respond to FeHm stress *in vitro* that did not fulfill criteria for inclusion as core modulon members. It is likely that such non-core FeHm-responsive genes will also be upregulated *in vivo*, and it is likely that more than just core genes are required for survival in the chinchilla ear. The core FeHm modulon contains 35 genes preferentially transcribed under FeHm-deplete conditions and 20 genes preferentially transcribed under FeHm-replete conditions. However, the total number of FeHm-regulated genes in all 5 studied isolates is approximately 250. It is likely that the pool of non-core genes allow adaptation to changing hosts and environmental niches within the host. It is known that *H. influenzae* is naturally transformable and shares genes between co-localized isolates. This is the fundamental principle of the Distributed Genome Hypothesis [31] and may in part explain why there is a large degree of heterogeneity of regulation seen between isolates *in vitro*, as well as variation in the genomic presence of various genes that are regulated in individual isolates. Future studies will focus on identification of all genes preferentially expressed *in vivo* with a view to obtaining a better understanding of the *in vivo* systems biology of *H. influenzae* disease.

## Methods

### Bacterial strains and growth conditions

NTHi strain 86-028NP is a nasopharyngeal isolate from a patient who underwent tympanostomy and tube insertion for chronic OM [32]. NTHi strain R2846 (originally designated strain 12) was isolated from the middle ear of a child with acute OM [33]. NTHi strain HI1722 (originally designated 1728MEE) was isolated from the middle ear of a child undergoing tympanostomy tube placement for chronic OM with effusion [27]. Isolates of *H. influenzae* were routinely maintained on chocolate agar with bacitracin at 37°C or grown in brain heart infusion (BHI) broth (Difco, Detroit, MI) supplemented with 10 μg/ml heme and 10 μg/ml β-NAD (supplemented BHI; sBHI). Heme-deplete growth was performed in BHI broth supplemented with 10 μg/ml β-NAD alone (heme-deplete BHI; hdBHI). Iron and heme restricted media (FeHm deplete) was hdBHI with deferoxamine (150 μM)

### Growth conditions for iron/heme regulated gene expression

Hemin was purchased from Sigma Chemical Co. (St. Louis, MO) and used to make stock heme solutions as previously described [34]. Growth conditions pertaining to the FeHm-regulation window of *H. influenzae* strains Rd KW20, 10810 and R2866 have been defined previously [24], and were used as the basis to define growth of NTHi strains R2846 and 86-028NP. For both experimental strains, various conditions were systematically evaluated to optimize growth characteristics, maintenance of viability consequent to FeHm starvation and reproducible regulation of gene expression. The following conditions were found to be optimal for the *in vitro* analysis of the regulation of gene transcription by iron and heme in strains 86-028NP and R2846. To prepare the primary inocula, *H. influenzae* were grown in 15 ml conical tubes containing 5 ml of BHI broth supplemented with 10 μg/ml β-NAD (BHI-NAD) and additionally supplemented with 2 μg/ml heme. These broth cultures were grown at 37°C on a rotator for 2 hours and were moderately turbid. To prepare the inocula, cells were pelleted by centrifugation, washed once in phosphate buffered saline (PBS) containing 0.1% gelatin and the pelleted cells were re-suspended in the same buffer. The suspension was adjusted to an A_605nm_ = 0.50 and diluted serially in the same buffer to provide an inoculum giving a final concentration of ~2 x 10^7^ cfu/ml when 5 ml of inoculum was added to 120 ml FeHm deplete BHI broth. Broth cultures for analysis of FeHm-mediated regulation of gene expression were incubated in a rotary shaker at 175 rpm at 37ºC, and 50 μl samples were removed at 30 minute intervals for determination of viable counts. For Q-PCR analyses, aliquots of 500 μl were removed at specified times and immediately mixed with 1 ml RNAProtect (Qiagen, Valencia, CA) and frozen at -70°C for later RNA preparation. Sixty milliliter samples for microarray studies were taken at 90 and 110 minutes of incubation, immediately mixed with 60 ml RNAProtect and stored frozen at -70°C for later RNA purification.

### RNA purification

Samples for Q-PCR obtained as described above were thawed, remixed by brief vortexing and incubated at room temperature for 5 minutes prior to purification using the RNeasy mini kit (Qiagen). Following purification, the sample was eluted with 40 μl of sterile RNase free water. Residual chromosomal DNA was removed by digestion with amplification grade DNase I (Invitrogen, Carlsbad, CA). The RNA samples were used to prepare cDNA as previously described [35]. Each 20 μl reaction contained 7 μl template RNA, 5.5 mM MgCl_2_, 500 μM each dNTP (dATP, dCTP, dGTP, dTTP), 1 x RT buffer, 80 mU RNase Inhibitor and 25 U MultiScribe Reverse Transcriptase (Applied Biosystems, Foster City, CA). The synthesis reaction was incubated at 25°C for 10 minutes followed by a further 30 minutes at 48°C. The reaction was terminated by heating at 95°C for 5 minutes. Prior to analysis, the cDNA was diluted by addition of 180 μl RNase-free water.

Samples for microarray obtained as described above were thawed and the cells collected by centrifugation. Total RNA was isolated using Trizol (Invitrogen) as described by the manufacturer. Residual genomic DNA was removed by treatment with RNase-free DNase (Invitrogen) as directed by the manufacturer and confirmed by Q-PCR analysis. The RNA samples were then subjected to LiCl precipitation as previously described [36] and concentrations determined using a Smartspec3000 (BioRad, Hercules, CA). Finally, to ensure that the RNA was not degraded, samples were resolved by PAGE using precast 6% TBE-urea gels (Invitrogen). On receipt by Nimblegen, each sample was subjected to additional quality control prior to processing for microarray analysis.

Samples from *in vivo* studies were prepared similarly to those for microarray. However, an additional step was added to the Trizol extraction. Fifty microliter samples of chinchilla effusions in RNA Protect (1:1) were added to 1 ml of Trizol. Prior to isopropanol precipitation, the top aqueous layer from the Trizol-chloroform extraction was subjected to a further phenol-chloroform-isoamyl alcohol, pH 6.6 (25:24:1) extraction to remove contaminants that were found to interfere with downstream enzymatic reactions.

### Quantitative real-time PCR

Q-PCR was performed as previously described [35]. Gene-specific oligonucleotide primers were designed using Primer Express 2.0 (Applied Biosystems) and synthesized by Operon Technologies (Huntsville, AL) and were tested to determine amplification specificity, efficiency and for linearity of the amplification with RNA concentration. Primers are listed in Additional file 5: Oligonucleotide primers used in this study. A typical 10 μl reaction contained 5 μl of SYBR Green Master Mix, 250 nM of each primer, and 2.5 μl of cDNA sample. Quantification reactions for the target transcripts at each timepoint were performed in quadruplicate and normalized to concurrently analyzed *gyrA* mRNA levels from the same sample. Relative quantification of gene expression was determined using the 2^-ΔΔCt^ method of Livak and Schmittgen where *ΔΔCt* = (*Ct*, *gene* - *Ct*, 16*s*)*time* * - (*Ct*, *gene* - *Ct*, 16*s*) *control*. [37].

### Microarray design

A microarray chip containing probes to all the genes of the NTHi isolates R2846, 86-028NP and R2866 as well as the Hib isolate 10810 was designed. The efficacy of this chip was demonstrated in a previous study [25]. Due to the frequency of phase variation in *H. influenzae* and the possibility of sequencing errors, all frame-shifted open reading frames were included on the arrays as a complete gene. Oligonucleotide probe sets for the array were designed by Nimblegen Systems, Inc. (Madison, WI). Each ORF of each genome is represented by thirteen longmer expression probes (60 nucleotides each). The probes were screened for uniqueness to minimize cross-hybridization. Each probe was replicated three times on each chip to increase accuracy.

Arrays were manufactured by Nimblegen Systems, Inc. by maskless array synthesis using a digital micro-mirror array-mediated, parallel synthesis process incorporating 5′-photoprotected phosphoramidites as previously described [38].

Post scan, the array features within the image file were extracted using NimbleScan v2.1. This program allows the user to combine the microarray image with the corresponding NimbleGen microarray design file, and optionally, with a gene description file to further map the image. The resulting alignment can be visually manipulated for further analysis. The Expression Data was processed using tools available through the Bioconductor project (http://www.bioconductor.org). Data was normalized using quantile normalization [39], and gene calls generated using the Robust Multichip Average (RMA) algorithm as described [40].

### Microarray data analysis

Technical array replicates (three duplicate probe sets were incorporated into each slide) were averaged prior to analysis of the three repeat experimental replicates of each isolate. The data were initially log_2_ transformed and compared between FeHm-replete and -deplete conditions by performing individual *t* tests using the TMEV software (http://www.tm4.org) [41]. Genes with a ≥1.5-fold expression change and *P* < 0.05 were considered significantly altered in gene expression.

### Genome sequencing of NTHi strain HI1722

The partial genome sequence of the NTHi strain HI1722 was obtained using the Applied Biosystems SOLiD V3.0 platform. A 10 μg sample of chromosomal DNA was sonicated with the Covaris S2 in order to generate fragments of 80–110 bp to be used for building fragment DNA libraries per existing SOLiD protocols (Foster City, CA). After shearing, DNA was end repaired and purified using PureLink PCR purification columns (Invitrogen) per manufacturer’s protocols. SOLiD sequencing adapters (P1 and P2) were ligated to the DNA fragments and the samples were run on agarose gels in order to size select and gel purify the 150–200 bp products followed by PCR amplification and nick translation for the adapter ligated products. Each DNA fragment library was column purified (Qiagen min-elute columns) and quantified using the Invitrogen Qubit fluorometer and broad range DNA assay. A standard amount of 60 pg for each library was used for separate emulsion PCR reactions (ePCR) following existing SOLiD protocols. Approximately 2.5 ×10^7^ beads were deposited for each sample onto a separate region of an octet slide for sequencing. Using the SOLiD V3.0, 50 bp sequencing reads were generated for each sample and resulting high quality reads were compared/aligned to the existing genome sequences of the *H. influenzae* strains Rd KW20, 86-028NP and 10810 to determine sequence homology using the SETS software tool that is integrated into the SOLiD platform. Additional reference alignments and/or assembly of orphan reads were processed using the CLC Genomics Workbench (CLC Bio USA, Cambridge, MA) software package and default parameters for *de novo* assembly.

### Chinchilla model of otitis media

A total of 7 adult chinchillas (*Chinchilla laniger*) with no evidence of middle ear infection by either otoscopy or tympanometry at the beginning of the study were used. Animals were rested for at least 7 days upon arrival to acclimate them to the vivarium. After acclimation, chinchillas were challenged with *H. influenzae* in two separate experiments. Animal procedures have been described in detail elsewhere [42-44].

In the first experiment five chinchillas were challenged in both ears transbullarly with approximately 2,000 CFU of NTHi strain 86-028NP. Transbullar inocula were delivered in 300 μl 0.1% gelatin in PBS by direct injection of bacterial suspensions into the superior bullae. The actual challenge dose was confirmed by plate count. On days 4, 7, 10, 14 and 17 post challenge middle ear effusions (MEE) were collected by epitympanic tap (i.e. withdrawl of fluids from the middle ear cavity using a 1.5 inch 25-gauge hypodermic needle) [44]. The majority of each recovered MEE was immediately mixed with an equal volume of RNAProtect and frozen in order to preserve the RNA profile for analysis by Q-PCR. A portion of each recovered MEE was reserved for determination of bacterial count using the track dilution method as previously described [45].

In the second experiment two chinchillas were challenged in both ears transbullarly with approximately 2,000 CFU of NTHi strain HI1722. Epitympanic taps were attempted on days 4, 7, 11, 14 and 18, after NTHI challenge. Recovered MEE were treated as described above.

This study was performed in strict accordance with the recommendations in the Guide for the Care and Use of Laboratory Animals (National Institutes of Health). Animal protocols were reviewed and approved by the Institutional Animal Care and Use Committee of the University of Oklahoma Health Sciences Center.

### Accession number

The microarray data from this study has been deposited with the Gene Expression Omnibus. The accession number is GSE38649.

## Competing interests

The authors declare that they have no competing interests.

## Authors’ contributions

All authors contributed to the design and execution of the experiments detailed. PWW, TWS, TMV performed microarray analysis. TWS performed growth studies. DJM performed chinchilla studies. TMV performed Q-PCR analyses. PWW drafted the manuscript. DJM, TWS and TLS revised the manuscript. All authors read and approved the final manuscript.

## Supplementary Material

Additional file 1 **Fold transcriptional change of 86-028NP genes following supplementation of FeHM-restricted media with exogenous FeHm.** The data compares fold transcriptional change of genes in *H. influenzae* strain 86-028NP in response to iron and heme supplementation of the growth media. The genes shown are only those that exhibit a significant change in the level of transcription. Click here for file

Additional file 2 **Fold transcriptional change of R2846 genes following supplementation of FeHm-restricted media with exogenous FeHm.** The data compares fold transcriptional change of genes in *H. influenzae* strain R2846 in response to iron and heme supplementation of the growth media. The genes shown are only those that exhibit a significant change in the level of transcription. Click here for file

Additional file 3 **Q-PCR values for FeHm core genes in 86-028NP infected chinchilla ears.** The data represent the Q-PCR values for the FeHm responsive core genes of *H. influenzae* strain 86-028NP in MEE samples from chinchillas infected with the specified strain. Click here for file

Additional file 4 **Q-PCR values for FeHm core genes in HI1722 infected chinchilla ears.** The data represent the Q-PCR values for the FeHm responsive core genes of *H. influenzae* strain HI1722 in MEE samples from chinchillas infected with the specified strain. Click here for file

Additional file 5 **Oligonucleotide primers used in this study.** This table lists all primers used for the Q-PCR analyses in the current study. Click here for file
