# Supplementary material for: Comparison of transcription of the Haemophilus influenzae iron/heme modulon genes in vitro and in vivo in the chinchilla middle ear
Source: BMC Genomics. 2013 Dec 27;14:925. doi: 10.1186/1471-2164-14-925 (PMC3879429; doi:10.1186/1471-2164-14-925)
Supplement: Additional file 1 — Fold transcriptional change of 86-028NP genes following supplementation of FeHM-restricted media with exogenous FeHm. The data compares fold transcriptional change of genes in H. influenzae strain 86-028NP in response to iron and heme supplementation of the growth media. The genes shown are only those that exhibit a significant change in the level of transcription. [file 1471-2164-14-925-S1.pdf]

Additional File 1. Fold transcriptional change of 86-086NP genes following supplementation of FeHm-restricted media with exogenous FeHm.

86-0268NP genes preferentially expressed in absence of FeHm

86-0286NP gene designation [a]	Description [b]	Rd KW20 locus [c]	ΔFC [d]
NTHI0088	Anaerobic ribonucleoside-triphosphate reductase, alpha subunit	HI0075	-1.66
NTHI0173	Hypothetical protein (truncated)	HI0094	-1.58
NTHI0175	Putative methyltransferase	HI0095	-3.47
NTHI0176	Hypothetical protein	HI0096	-3.73
NTHI0177	Iron(III) ABC transporter periplasmic binding protein HitA	HI0097	-2.38
NTHI0179	Iron(III) ABC transporter permease protein HitB	HI0098	-2.24
NTHI0180	Iron(III) ABC transporter ATP-binding protein HitC	HI0099	-2.03
NTHI0181	Conserved hypothetical protein	HI0101m	-1.52
NTHI0202	Probable TonB-dependent heme receptor	HI0113	-1.64
NTHI0242	Putative anaerobic C4-dicarboxylate transporter	HI0153	-1.54
NTHI0359	Biopolymer transport protein ExbD	HI0252	-1.76
NTHI0360	Biopolymer transport protein ExbB	HI0253	-1.84
NTHI0369	Heme-hemopexin utilization protein HxuC	HI0262	-2.64
NTHI0370	Heme-hemopexin utilization protein HxuB	HI0263	-3.93
NTHI0371	Heme-hemopexin utilization protein HxuA	HI0264	-2.11
NTHI0372	Dihydroneopterin aldolase	HI0265	-1.52
NTHI0477	Fe/Mn/Zn ABC transporter permease protein	HI0359	-1.66
NTHI0478	Fe/Mn/Zn ABC transporter permease protein	HI0360	-1.81
NTHI0479	Fe/Mn/Zn ABC transporter ATP-binding protein	HI0361	-2.12
NTHI0481	Fe/Mn/Zn ABC transporter periplasmic-binding protein	HI0362	-1.78
NTHI0492	[2FE-2S] ferredoxin, electron carrer protein	HI0372	-1.69
NTHI0630	D-ribose ABC transporter, ATP-binding protein	HI0502	-2.03
NTHI0631	D-ribose ABC transporter, permease protein	HI0503	-1.65
NTHI0660	Aspartate-ammonia lyase AspA	HI0534	-1.67
NTHI0743	Conserved hypothetical protein	n/a	-1.54
NTHI0782	Hemoglobin-haptoglobin binding protein HgpB	HI0661	-3.09
NTHI0783	Putative glutathione ABC transporter, fused ATPase and permease	HI0663m	-2.88
NTHI0785	Putative glutathione ABC transporter, fused ATPase and permease	HI0664	-2.46
NTHI1168	Transferrin-binding protein 1	HI0994	-3.15
NTHI1169	Transferrin-binding protein 2	HI0995	-6.92
NTHI1171	Putative outer membrane protein OmpU	HI0997m	-7.44
NTHI1172	50S ribosomal protein L34	HI0998	-4.37
NTHI1206	Anaerobic dimethyl sulfoxide reductase, subunit B	HI1046	-2.45
NTHI1207	Anaerobic dimethyl sulfoxide reductase, subunit A	HI1047	-2.78

NTHI1503	Hypothetical protein	n/a	-1.59
NTHI1707	Putative ABC transporter	HI1427	-1.52
NTHI1794m	Probable TonB-dependent transport protein	HI1369	-1.7
NTHI1809	1,4-alpha-glucan branching enzyme	HI1357	-1.65
NTHI1810	4-alpha-glucanotransferase (amylomaltase)	HI1356	-1.76

86-0286NP Genes preferentially expressed in presence of FeHm

86-0286NP gene designation	Description	Rd KW20 locus	ΔFC
NTHI0007	Formate dehydrogenase, major subunit	HI0006m (fs)	1.71
NTHI0010	Formate dehydrogenase-N, Fe-S beta subunit	HI0007	1.73
NTHI0011	Formate dehydrogenase-N, cytochrome B556(Fdn) γ subunit	HI0008	1.76
NTHI0012	Formate dehydrogenase formation protein FdhE	HI0009	1.75
NTHI0013	Acyltransferase for 30S ribosomal subunit protein S18	HI0010	1.59
NTHI0276m	Alcohol dehydrogenase class III	HI0185	2.85
NTHI0278	Putative transcriptional regulator	HI0186	1.78
NTHI0462	Periplasmic nitrate reductase assembly protein NapD	HI0343	1.6
NTHI0463	Periplasmic nitrate reductase, subunit A	HI0344	1.62
NTHI0464	Putative ferredoxin-type protein NapG	HI0345	1.91
NTHI0465	Putative ferredoxin-type protein NapH	HI0346	1.71
NTHI0466	Periplasmic nitrate reductase, small subunit B	HI0347	2.17
NTHI0467	Periplasmic nitrate reductase, cytochrome C-type protein NapC	HI0348	2.34
NTHI1209	Putative heavy metal transport protein	HI1049m	1.64
NTHI1211	Putative heavy metal chaperone protein	HI1050	2.3
NTHI1227	Nitrite reductase complex protein NrfC	HI1067	3.53
NTHI1229	Nitrite reductase complex protein NrfB	HI1068	3.81
NTHI1230	Nitrite reductase complex, cytochrome C552 subunit	HI1069	3.65
NTHI1231	Probable ATP-dependent RNA helicase	HI1070	1.6
NTHI1255	Cytochrome c biogenesis cluster: heme export ABC transporter, heme binding protein	HI1091	1.67
NTHI1256	Cytochrome c biogenesis cluster: heme export ABC transporter, CcmD component	HI1092	1.68
NTHI1258	Cytochrome c biogenesis cluster: heme lyase protein F	HI1094	1.65
NTHI1259	Cytochrome c biogenesis cluster: heme lyase/disulfide oxidoreductase (DsbE)	HI1095	1.52
NTHI1773	Ferritin protein A1	HI1384	2.06
NTHI1772	Ferritin protein A2	HI1385	2.23

a. Locus identifier. Numbering refers to the 86-028NP annotation of CDS

b. Predicted gene function

c. Homologous locus in *H. influenzae* Rd KW20.

d. Fold difference change in expression level.
