# Supplementary material for: Comparison of transcription of the Haemophilus influenzae iron/heme modulon genes in vitro and in vivo in the chinchilla middle ear
Source: BMC Genomics. 2013 Dec 27;14:925. doi: 10.1186/1471-2164-14-925 (PMC3879429; doi:10.1186/1471-2164-14-925)
Supplement: Additional file 2 — Fold transcriptional change of R2846 genes following supplementation of FeHm-restricted media with exogenous FeHm. The data compares fold transcriptional change of genes in H. influenzae strain R2846 in response to iron and heme supplementation of the growth media. The genes shown are only those that exhibit a significant change in the level of transcription. [file 1471-2164-14-925-S2.pdf]

Additional File 2. Fold transcriptional change of R2846 genes following supplementation of FeHm-restricted media with exogenous FeHm.

NTHi R2846 Genes preferentially expressed in absence of FeHm

NTHi R2846 gene designation [a]	Description [b]	Rd KW20 locus [c]	ΔFC [d]
r2846v6.53	Aspartate-ammonia lyase AspA	HI0534	-2.62
r2846v6.66	Conserved hypothetical protein	HI0521	-1.56
r2846v6.81c	Ribose operon repressor	HI0506	-2.99
r2846v6.82c	Ribokinase	HI0505	-2.19
r2846v6.83c	D-ribose ABC transporter, periplasmic-binding protein	HI0504	-12.57
r2846v6.84c	D-ribose ABC transporter, permease protein	HI0503	-15.16
r2846v6.85c	D-ribose ABC transporter, ATP-binding protein	HI0502	-22.15
r2846v6.86c	D-ribose pyranase	HI0501	-42.36
r2846v6.93	AphA - acid phosphatase	HI0495m	-1.83
r2846v6.216	transcriptional regulator IscR	HI0379	-1.66
r2846v6.220	Fe-S cluster co-chaperone protein HscB	HI0375	-1.68
r2846v6.235	Fe/Mn/Zn ABC transporter periplasmic-binding protein	HI0362	-1.57
r2846v6.236	Fe/Mn/Zn ABC transporter ATP-binding protein	HI0361	-1.74
r2846v6.312	Copper chaperone protein	HI0292	-1.53
r2846v6.315	Probable serine transporter	HI0289	-1.76
r2846v6.316	L-serine deaminase	HI0288	-1.51
r2846v6.340c	Heme-hemopexin utilization protein HxuA	HI0264	-2.55
r2846v6.341c	Heme-hemopexin utilization protein HxuB	HI0263	-2.92
r2846v6.342c	Heme-hemopexin utilization protein HxuC	HI0262	-3.95
r2846v6.352	Biopolymer transport protein ExbB	HI0253	-1.64
r2846v6.353	Biopolymer transport protein ExbD	HI0252	-1.54
r2846v6.401	Hypothetical protein	HI0223	-1.54
r2846v6.409	Adenylate cyclase	HI0604	-1.72
r2846v6.413	Probable transport permease	HI0608	-1.51
r2846v6.505	Putative anaerobic C4-dicarboxylate transporter	HI0153	-2
r2846v6.513c	Putative TRAP transport system, periplasmic-binding component	HI0146	-1.53
r2846v6.540c	Probable TonB-dependent heme receptor	HI0113	-3.39
r2846v6.560c	Iron(III) ABC transporter ATP-binding protein HitC	HI0099	-2.19
r2846v6.561c	Iron(III) ABC transporter permease protein HitB	HI0098	-2.33
r2846v6.563c	Iron(III) ABC transporter periplasmic binding protein HitA	HI0097	-2.39
r2846v6.565	Putative methyltransferase	HI0095	-2.15
r2846v6.585c	Anaerobic ribonucleoside-triphosphate reductase, alpha subunit	HI0075	-2.34
r2846v6.627	Conserved hypothetical protein/predicted permease component	HI0035	-2.16
r2846v6.822c	Tellurite resistance protein TehB	HI1275	-2.34

r2846v6.866	4-alpha-glucanotransferase (amylomaltase)	HI1356	-2.29
r2846v6.867	1,4-alpha-glucan branching enzyme	HI1357	-2.44
r2846v6.868	Glycosyl hydrolase	HI1358	-2.35
r2846v6.869	Glucose-1-phosphate adenylyltransferase	HI1359	-2.32
r2846v6.870	Glycogen synthase	HI1360	-2.03
r2846v6.881c	Probable TonB-dependent transport protein	HI1369	-8.04
r2846v6.938	Anaerobic regulatory protein Fnr	HI1425	-1.61
r2846v6.940	Putative ABC transporter	HI1427	-2.65
r2846v6.1043	HI1466m (putative OMP truncated)	HI1466m	-4.43
r2846v6.1165c	Nickel and cobalt ABC transporter, periplasmic binding protein	HI1624	-1.52
r2846v6.1202c	Malate dehydrogenase	HI1210	-1.97
r2846v6.1224	Conserved hypothetical protein	HI1190	-1.65
r2846v6.1371	Anaerobic dimethyl sulfoxide reductase, subunit A	HI1047	-2.01
r2846v6.1372	Anaerobic dimethyl sulfoxide reductase, subunit B	HI1046	-1.71
r2846v6.1407c	Putative outer membrane protein OmpU	HI0997m	-9.24
r2846v6.1408	Transferrin-binding protein 2	HI0995	-8.57
r2846v6.1409	Transferrin-binding protein 1	HI0994	-8.43
r2846v6.1615	Galactose-1-phosphate uridylyltransferase	HI0820	-1.59
r2846v6.1628	Phosphoenolpyruvate carboxykinase	HI0809	-1.92
r2846v6.1670c	3,4-dihydroxy-2-butanone 4-phosphate synthase	HI0764	-1.55
r2846v6.1693c	L-asparaginase II	HI0745	-2.6
r2846v6.1732	Hemoglobin-haptoglobin binding protein HgpC	HI0712	-2.02
r2846v6.1764c	Glycerol kinase	HI0691	-1.58
r2846v6.1765c	Glycerol uptake facilitator protein	HI0690	-1.56
r2846v6.1773	putative ferric hydroxamate uptake protein FhuC	n/a	-2.85
r2846v6.1774	putative ferric hydroxamate uptake protein FhuD	n/a	-3.07
r2846v6.1775	putative ferric hydroxamate uptake protein FhuB	n/a	-2.95
r2846v6.1777	Probable TonB-dependent receptor	n/a	-2.97
r2846v6.1778	Putative transposase remnant	n/a	-2.6
r2846v6.1794	Hemoglobin-haptoglobin binding protein HgpB	HI0661	-2.51
r2846v6.1814	Trimethylamine N-oxide reductase system III, cytochrome c-type subunit	HI0644	-2.28
r2846v6.1815	Trimethylamine N-oxide reductase system III, catalytic subunit	HI0643	-2.05
r2846v6.1846c	DNA transformation regulatory protein	HI0601	-1.51
r2846v6.1856	Peptidase E	HI0587	-1.67
r2846v6.1857	Putative C4 dicarboxylate transporter	HI0586m	-1.65
r2846v6.1859	Putative peptidase/hydrolase	HI0584	-1.75

R2846 Genes preferentially expressed in presence of FeHm

NTHi R2846 gene designation	Description	Rd KW20 locus	ΔFC

r2846v6.248c	Periplasmic nitrate reductase, cytochrome C-type protein NapC	HI0348	1.69
r2846v6.249c	Periplasmic nitrate reductase, small subunit B	HI0347	1.68
r2846v6.250c	Putative ferredoxin-type protein NapH	HI0346	1.86
r2846v6.251c	Putative ferredoxin-type protein NapG	HI0345	2.13
r2846v6.252c	Periplasmic nitrate reductase, subunit A	HI0344	1.79
r2846v6.253c	Periplasmic nitrate reductase assembly protein NapD	HI0343	1.78
r2846v6.469	Alcohol dehydrogenase class III	HI0185	12.18
r2846v6.470	Conserved hypothetical protein	HI0184	15.83
r2846v6.898	Ferritin protein A1	HI1384	1.62
r2846v6.899	Ferritin protein A2	HI1385	1.58
r2846v6.901	Hypothetical protein	HI1386m	1.87
r2846v6.960	5,10-methylenetetrahydrofolate reductase	HI1444	1.62
r2846v6.1263c	Predicted Na+/dicarboxylate symporter	HI1154	1.57
r2846v6.1326c	Cytochrome c biogenesis cluster: heme lyase protein F	HI1094	1.63
r2846v6.1340	Probable amino acid ABC transporter periplasmic-binding protein	HI1080	2.54
r2846v6.1341	Probable amino acid ABC transporter permease component	HI1079m	2.48
r2846v6.1342	Probable amino acid ABC transporter ATP-binding protein	HI1078	2.21
r2846v6.1351	Nitrite reductase complex, cytochrome C552 subunit	HI1069	5.11
r2846v6.1352	Nitrite reductase complex protein NrfB	HI1068	4.75
r2846v6.1353	Nitrite reductase complex protein NrfC	HI1067	2.98
r2846v6.1354	Nitrite reductase complex protein NrfD	HI1066	3.84
r2846v6.1369c	Putative heavy metal transport protein	HI1049m	1.52
r2846v6.1370c	Conserved hypothetical protein	HI1048	1.52
r2846v6.1423c	DNA architectural protein Fis	HI0980	1.66

a. Locus identifier. R2846v6 refers to the file used for annotation and the number to the CDS

b. Predicted gene function

c. Homologous locus in Hi Rd KW20. n/a Not applicable, no homologous loci.

d. Fold difference change in expression level.
