# Supplementary material for: Comparison of transcription of the Haemophilus influenzae iron/heme modulon genes in vitro and in vivo in the chinchilla middle ear
Source: BMC Genomics. 2013 Dec 27;14:925. doi: 10.1186/1471-2164-14-925 (PMC3879429; doi:10.1186/1471-2164-14-925)
Supplement: Additional file 3 — Q-PCR values for FeHm core genes in 86-028NP infected chinchilla ears. The data represent the Q-PCR values for the FeHm responsive core genes of H. influenzae strain 86-028NP in MEE samples from chinchillas infected with the specified strain. [file 1471-2164-14-925-S3.pdf]

Additional File 3. Q-PCR values for FeHm core genes in 86-028NP infected chinchilla ears

	Chinchilla ear sample[a]																	
Locus[b]	2LD7	2LD10	2L14	2RD7	2RD10	2RD14	2RD17	4LD7	4LD10	4RD7	4RD10	4RD14	4RD17	5LD7	5LD10	5LD14	5LD17	5RD14
HI0007	-1.01	-1.13	-1.04	1.21	1.50	1.32	1.89	1.01	1.06	1.47	1.22	1.33	1.74	-1.19	1.00	1.10	1.24	-1.49
HI0035	14.08	24.81	10.83	15.48	14.56	11.28	21.52	18.12	12.83	16.28	17.30	11.02	11.34	27.01	17.83	20.27	10.85	15.41
HI0075	3.55	2.39	5.16	4.31	3.56	4.41	3.79	4.04	4.99	5.21	3.56	4.34	5.58	3.16	2.76	3.15	3.24	3.27
HI0095	6.67	5.59	8.24	7.61	8.63	8.30	5.96	9.13	8.91	7.22	6.93	7.74	9.12	4.34	5.28	5.82	7.80	7.26
HI0097	3.28	5.67	1.34	5.03	3.51	1.18	3.74	7.22	5.85	1.66	2.54	1.41	1.25	1.47	1.34	1.63	1.28	2.47
HI0113	1.36	1.52	5.76	2.58	1.34	4.50	-1.92	1.27	3.65	3.87	4.95	5.44	4.03	2.96	3.11	1.59	4.78	-1.13
HI0153	6.57	6.59	6.74	6.93	7.52	6.52	8.29	9.15	8.85	9.15	5.79	6.23	7.52	7.48	5.31	7.88	6.68	7.21
HI0185	1.14	-1.46	-1.68	-1.32	-1.84	-2.27	1.51	-1.02	-1.39	-1.78	-1.73	-2.10	-2.21	1.15	-1.34	-1.36	-1.45	-1.43
HI0253	-2.27	-3.10	-1.81	-2.89	-3.14	-2.11	-3.69	-2.50	-2.01	-2.05	-2.24	-1.93	-1.96	-2.17	-2.16	-2.71	-2.43	-3.91
HI0263	4.87	9.60	3.78	6.13	5.86	7.79	5.11	3.18	5.11	7.31	6.65	7.98	10.20	6.86	5.86	4.36	7.58	4.04
HI0343	1.34	-1.02	2.19	1.58	-1.01	1.97	-1.54	1.23	1.94	2.57	1.82	1.88	2.20	1.58	1.95	1.34	1.95	-1.20
HI0362	3.67	2.29	5.87	1.98	1.93	1.77	1.15	1.70	2.85	1.67	1.95	1.59	1.80	1.46	1.58	2.10	2.09	4.97
HI0534	4.42	5.08	9.53	7.84	6.09	8.77	4.48	5.77	9.16	8.31	7.91	9.02	9.17	5.07	5.82	5.74	7.41	5.73
HI0584	1.74	1.64	3.99	3.05	2.08	3.50	1.81	2.09	2.69	3.16	2.89	3.77	3.48	2.38	2.25	2.02	3.10	1.60
HI0661	7.43	6.35	5.99	2.29	3.28	2.59	2.02	2.63	2.44	2.42	2.74	2.27	2.57	1.79	2.46	2.16	2.96	5.38
HI0691	1.12	-1.04	1.08	1.14	1.40	1.54	1.80	1.23	1.56	1.21	1.11	1.25	1.51	-1.26	-1.23	1.33	1.12	1.42
HI0809	1.04	1.37	1.12	1.25	1.42	1.45	1.66	1.29	2.05	1.51	1.72	1.42	1.59	2.04	1.13	1.41	1.31	-1.15
HI0980	1.37	-1.27	1.81	1.60	1.27	2.64	1.56	1.02	1.82	2.49	2.32	2.17	2.66	1.47	1.44	1.33	2.11	-1.03
HI0994	3.68	2.14	6.33	2.55	2.05	4.80	1.10	1.36	2.80	5.67	6.45	7.84	7.07	7.96	5.97	2.88	7.63	1.19
HI0997m	31.60	64.48	21.40	45.68	44.09	60.01	44.73	33.17	33.03	54.43	47.37	55.23	78.02	30.45	37.07	32.34	60.70	30.30
HI1069	2.82	2.84	1.83	2.16	2.81	1.96	4.04	2.94	3.24	1.85	1.33	1.81	2.03	2.11	1.62	2.93	1.72	2.71
HI1078	2.25	2.54	3.04	3.86	1.93	2.33	n/a	5.01	5.33	2.50	1.17	2.16	n/a	-1.11	2.12	-1.12	n/a	n/a
HI1094	15.02	17.39	6.93	12.58	17.89	7.93	17.63	15.35	9.80	8.50	5.77	6.13	7.54	5.81	5.95	11.10	6.30	13.22
HI1210	1.84	1.54	3.36	2.72	2.51	3.30	6.80	2.74	3.70	2.50	2.10	2.74	3.82	1.44	1.27	2.008	2.65	3.18
HI1356	8.80	4.52	16.97	9.24	8.29	16.03	6.30	6.97	10.76	15.14	12.64	15.50	18.18	8.37	11.52	8.65	14.01	6.53
HI1369	21.10	18.31	38.86	28.35	28.28	36.27	19.92	17.36	38.51	40.48	41.76	39.49	41.77	52.45	38.56	23.43	34.65	17.01
HI1384	-7.19	-9.35	-3.44	-10.10	-16.95	-6.33	-62.50	-14.29	-7.09	-6.33	-5.32	-4.15	-4.74	-3.61	-5.32	-9.80	-3.88	-17.86
HI1427	9.85	11.12	6.79	7.45	9.14	8.09	8.68	10.07	12.31	8.34	10.95	7.78	8.86	7.32	7.19	10.47	6.57	7.47

[a] Designation refers to animal, ear and day of sample. The first number refers to the animal, L or R to the left or right ear respectively and D*n* indicates the day of sampling. Thus 2LD7 is Animal 2, left ear, day 7.

[b.] Gene locus in Rd KW20
