# Supplementary material for: Comparison of transcription of the Haemophilus influenzae iron/heme modulon genes in vitro and in vivo in the chinchilla middle ear
Source: BMC Genomics. 2013 Dec 27;14:925. doi: 10.1186/1471-2164-14-925 (PMC3879429; doi:10.1186/1471-2164-14-925)
Supplement: Additional file 4 — Q-PCR values for FeHm core genes in HI1722 infected chinchilla ears. The data represent the Q-PCR values for the FeHm responsive core genes of H. influenzae strain HI1722 in MEE samples from chinchillas infected with the specified strain. [file 1471-2164-14-925-S4.pdf]

Additional File 4. Q-PCR values for FeHm core genes in HI1722 infected chinchilla ears

	Chinchilla ear sample[a]					
Locus[b]	2RD4	2RD7	1RD4	1RD7	1RD11	1RD14
HI0007	2.48	2.20	-1.04	1.10	2.01	3.07
HI0035	34.20	42.85	11.03	14.34	34.47	80.41
HI0075	17.74	15.46	34.15	19.12	14.63	35.70
HI0095	14.26	20.11	28.91	21.36	18.92	33.97
HI0097	12.55	7.45	228.99	15.17	15.71	34.35
HI0113	6.69	6.03	5.03	5.00	4.46	8.55
HI0153	5.72	2.97	4.86	9.01	4.08	9.81
HI0185	-1.09	-1.07	-1.94	-5.1	-1.04	1.45
HI0253	30.10	23.65	73.68	91.33	21.28	28.35
HI0263	16.07	10.79	17.31	15.14	10.93	16.41
HI0343	5.94	4.88	20.05	14.38	19.93	21.82
HI0362	47.21	13.81	226.07	276.32	40.79	175.87
HI0534	44.30	31.27	27.66	22.42	41.42	68.67
HI0584	11.10	13.46	8.26	5.69	10.48	14.01
HI0661	38.11	115.45	262.69	233.60	155.18	111.89
HI0691	3.70	4.02	2.14	3.72	5.99	8.57
HI0809	40.02	9.59	44.77	48.78	19.32	43.86
HI0980	1.69	1.44	5.28	6.24	1.27	1.68
HI0994	31.55	36.45	22.83	18.31	24.16	44.27
HI0997m	47.86	40.16	42.85	16.69	50.46	12.09
HI1069	17.67	10.09	3.33	5.60	10.90	22.80
HI1078	4.55	7.38	1.47	1.12	5.85	9.11
HI1094	3.85	7.09	3.64	2.50	6.80	6.23
HI1210	2.03	2.43	4.57	4.39	3.03	6.16
HI1356	15.43	25.37	4.81	2.64	22.99	14.39
HI1369	56.51	24.42	35.86	83.04	24.50	8.21
HI1384	-4.46	-3.79	-4.46	-4.08	-6.13	-13.33
HI1427	3.85	3.32	24.60	20.50	4.58	5.64

[a] Designation refers to animal, ear and day of sample. The first number refers to the animal, L or R to the left or right ear respectively and D*n* indicates the day of sampling. Thus 2RD4 is Animal 2, right ear, day 4.

[b.] Gene locus in Rd KW20
