# Supplementary material for: Comparison of transcription of the Haemophilus influenzae iron/heme modulon genes in vitro and in vivo in the chinchilla middle ear
Source: BMC Genomics. 2013 Dec 27;14:925. doi: 10.1186/1471-2164-14-925 (PMC3879429; doi:10.1186/1471-2164-14-925)
Supplement: Additional file 5 — Oligonucleotide primers used in this study. This table lists all primers used for the Q-PCR analyses in the current study. [file 1471-2164-14-925-S5.pdf]

Additional File 5. Oligonucleotide primers used in this study			
Primer	Gene	Target strain(s)	Sequence
QPCR-HI0007-uF	*fdnH*	All	5’-GATCGTTTAGAATGGCTGATTCG
QPCR-HI0007-uR	*fdnH*	All	5’-TGCAGGACAAGCCTTTAAACAA
QPCR-HI0035-F1		All	5’-GGGCAGGGCAGCAAATT
QPCR-HI0035-uR		All	5’-GCGGATTAACCACATTGCAA
QPCR-HI0075-uF	nrdD	All	5’-TGGGCAAACACCATTTGTGA
QPCR-HI0075-R1	nrdD	All	5’-TTGCTGAATTAAGCGGGATTG
QPCR-HI0095-uF		All	5’-TTGCGTCCAGGCGGTAA
QPCR-HI0095-R1		All	5’-ACCTCTAACACTTTTTTATCTTGGCTAAA
QPCR-HI0097-uF	*hitA*	All	5’-CAGGGCTTTTAGCACCAATTTC
QPCR-HI0097-uR	*hitA*	All	5’-ACGGCCACTTAATGCAATCC
QPCR-HI0113-uF	*hemR*	All	5’-TGTAACCGCAAAACAAAATTCTTC
QPCR-HI0113-uR	*hemR*	All	5’-TGTTTTAATACTGAACGACCAGGTAGTTTAT
QPCR-HI0153-uF	*dcuB*	All	5’-TTGCGCATCCGTACTTGAAG
QPCR-HI0153-uR	*dcuB*	All	5’-CGGAGAATGCGTTCAGCAAT
QPCR-HI0185-F2	*adhC*	All	5’-CTGCTGAATGTGGCGAATGT
QPCR-HI0185-R1	*adhC*	86028, 1722	5’- CGTGCAATCAGGCATTAAGC
QPCR-HI0185-R3	*adhC*	R2846	5’-CGTACCATCTGGCATTAAGC
QPCR-HI0253-F2	*exbB*	All	5’-CCAAGTGTAATTATGTTGCACTTATCT
QPCR-HI0253-uR	*exbB*	All	5’-CGCCCCAAACCGTTATAAAA
QPCR-HI0263-uF	*hxuB*	All	5’-ACCTCGTTATTCTGTTATTGCAAGTG
QPCR-HI0263-R1	*hxuB*	All	5’-CACGGTTCAATGATCCAGTATCTG
QPCR-HI0343-uF	*napD*	All	5’-TCGTGCAAGGTAATCCAAAAAA
QPCR-HI0343-uR	*napD*	All	5’-GTCGGAATTTCAGTATGCTCAATC
QPCR-HI0362-uF	*yfeA*	All	5’-AATGCCGCTGACTATGCTCAA
QPCR-HI0362-R1	*yfeA*	All	5’-GCGCTTCTGGAATTTGTGCTA
QPCR-HI0534-uF	*aspA*	All	5’-AAGTGATATTGCAAAAGCGATTGTA
QPCR-HI0534-R2	*aspA*	All	5’-ACATCTGATGGGAATTGATCTAAGC
QPCR-HI0584-uF		All	5’-ACGATGGTTTTATGCATGCTTGT
QPCR-HI0584-uR		All	5’-GCGATCCATAATGCAACACCTAA
QPCR-HI0661-F1	*hgpB*	All	5’-CTGTTGTTGAAGCTGGACGTTTT
QPCR-HI0661-uR	*hgpB*	All	5’-TTCAGCTTGACGTAATCCATCAAT
QPCR-HI0691-uF	*glpK*	All	5’-ACGCGATCGTTTGGCAAT
QPCR-HI0691-uR	*glpK*	All	5’-TGGCCATCGGCTTTTAATTT
QPCR-HI0809-uF	*pckA*	All	5’-TGGTACGGCGGCGAAAT
QPCR-HI0809-uR	*pckA*	All	5’-GCAGTGCATTGCACCAACA
QPCR-HI0980-uF	*fis*	All	5’-TCAGTATTAAATGCGCAATCACAA
QPCR-HI0980-R2	*fis*	All	5’-AATAATTACGCAACGCTTGTTTGA
QPCR-HI0994-uF	*tbp1*	All	5’-AAGTAAGAGATCGTAAAGATAATGAAGTAACTG
QPCR-HI0994-uR	*tbp1*	All	5’-ACCGCGACCTTGTTCTACAACT
QPCR-HI0997m-F3	*ompU1*	86028, 1722	5’-AACGAATATAGCTTGGGCAAAAGTT
QPCR-HI0997m-F4	*ompU1*	R2846	5’-AACGAATGTAGCTTGGGCAGAAGTT
QPCR-HI0997m-uR	*ompU1*	All	5’-GAGGTTTTTAATTCCGCACTTTGA
QPCR-HI1069-uF	*nrfA*	All	5’-TGCAAATGATGGCCCTCAA
QPCR-HI1069-uR	*nrfA*	All	5’-GCGATTAAACGTGGAACATCAG
QPCR-HI1078-uF	*tcyC*	All	5’-GGGCTTAAAAATAAAGCGGATTTA
QPCR-HI1078-uR	*tcyC*	All	5’-CAATCCCCACTCGCTGTTG
QPCR-HI1094-uF	*ccmF*	All	5’-GGGATCCAGTAGAAAACTCATCGT
QPCR-HI1094-uR	*ccmF*	All	5’-CTGTCACAGAAAGAGAGTGAATCAATG
QPCR-HI1210-F1	*mdh*	86028, 1722	5’-AAGTTGCTGTATTAGGTGCCGC
QPCR-HI1210-F2	*mdh*	R2846	5’-AAGTTGCTGTATTAGGAGCCGC
QPCR-HI1210-uR	*mdh*	All	5’-AATAACGCTAATGCTTGACCAATACC
QPCR-HI1356-uF	*malQ*	All	5’-ATCCTCGAAATGCTTATGCCACTA
QPCR-HI1356-R2	*malQ*	All	5’-TAATTCTAAATCACGGCAATGCCAAA
QPCR-HI1369-uF		All	5’-TCGCACGACAAATTCCTCTTG

QPCR-HI1369-R1		R2846	5’-TGTAACTCTGCCACTCCGACAT
QPCR-HI1369-R2		86028	5’-TGTAGCTCTGCCACTCCGACAA
QPCR-HI1369-R4		1722	5’-TGTAACTCTGCCGCACCAGAAT
QPCR-HI1384-uF	*ftnA*	All	5’-GCTGAACGACCAAATTAACTTAGAGTT
QPCR-HI1384-uR	*ftnA*	All	5’-GCATGACGAAGTAAGAAGGTAGCA
QPCR-HI1427-F1		R2846	5’-AGAGTTGGTTTACCGCTAGAAATTG
QPCR-HI1427-F2		86028, 1722	5’-AGAGTTGGTTTACCACTGGAAATTG
QPCR-HI1427-uR		All	5’-ACGGTTCGCCTGATTTCG
